# Increased financial burden among patients with chronic myelogenous leukaemia receiving imatinib in Japan: a retrospective survey

**DOI:** 10.1186/1471-2407-12-152

**Published:** 2012-04-24

**Authors:** Yuko Kodama, Ryoko Morozumi, Tomoko Matsumura, Yukiko Kishi, Naoko Murashige, Yuji Tanaka, Morihito Takita, Nobuyo Hatanaka, Eiji Kusumi, Masahiro Kami, Akihiko Matsui

**Affiliations:** 1 Division of Social Communication System for Advanced Clinical Research, the Institute of Medical Science, University of Tokyo, 4-6-1, Shirokanedai, Minato-ku, Tokyo, 108-8639, Japan; 2 Faculty of Economics, University of Toyama, 3190 Gakufu, Toyama-shi, Toyama, 930-8555, Japan; 3 Faculty of Economics, The University of Tokyo, 7-3-1, Hongo, Bunkyo-ku, Tokyo, 113-0033, Japan

**Keywords:** Economic recession, Hematology, Anticancer drug, Health insurance

## Abstract

****Background**:**

The financial burden of medical expenses has been increasing for cancer patients. We investigated the relationship between household income and financial burden among patients with chronic myelogenous leukaemia (CML) who have been treated with imatinib.

****Methods**:**

A questionnaire was distributed to 1200 patients between May and August 2009. We retrospectively surveyed their household incomes, out-of-pocket medical expenses, final co-payments after refunds, and the perceived financial burden of their medical expenses in 2000, 2005 and 2008.

****Results**:**

A total of 577 patients completed the questionnaire. Their median age was 61 years (range, 15–94). A financial burden was felt by 41.2 % (28 of 68) of the patients treated with imatinib in 2000, 70.8 % (201 of 284) in 2005, and 75.8 % (400 of 528) in 2008. Overall, 182 patients (31.7 %) considered its discontinuation because of the financial burden and 15 (2.6 %) temporarily stopped their imatinib prescription. In 2000, 2005 and 2008, the patients’ median annual household incomes were 49,615 US Dollars (USD), 38,510 USD and 36,731 USD, respectively, with an average currency exchange rate of 104 Yen/USD in 2008. Their median annual out-of-pocket expenses were 11,548, 12,067 and 11,538 USD and their median final annual co-payments were 4,375, 4,327 and 3,558 USD, respectively. Older patients (OR = 0.96, 95 % CI: 0.95–0.98, *p* ≪ 0.0001 for 1-year increments), and patients with higher household incomes (OR = 0.92, 95 % CI: 0.85–0.99, *p* = 0.03 for 10,000 USD-increments) were less likely to have considered discontinuing their imatinib treatment. Conversely, patients with higher annual final co-payments (OR = 2.21, 95 % CI: 1.28–4.28, *p* = 0.004 for 10,000 USD-increments) were more likely to have considered discontinuing their imatinib treatment.

****Conclusions**:**

The proportion of CML patients who sensed a financial burden increased between 2000 and 2008. During this period, their annual incomes fell by 13,000 USD, although their medical expenses did not change. Financial support for patients being treated with expensive drugs remains a major problem in Japan.

## **Background**

Following the global financial crisis triggered by the collapse of Lehman Brothers in 2008, many developed countries have faced a severe economic recession. The Japanese economy has consequently shown signs of further stagnation. Along with a nationwide recession, household income in Japan has declined since 2000. Japan’s financial problems have in fact been worsened by the recent earthquake, tsunami, and Fukushima nuclear crisis of March 2011. Meanwhile, because of population-aging, social welfare costs have accounted for higher proportions of government spending in developed countries than ever before. To maintain the financial balance of their insurance systems, governmental agencies in many nations carefully review all applications for approval of expensive medications, such as anticancer drugs [1-3].

Japan established a universal public medical insurance system in 1961. By law, all Japanese citizens must join public health insurance programs and can access all hospitals, regardless of income or insurance policy [4]. This system has been frequently revised to adapt to social changes [4]. At present, all patients have to pay a part of their medical costs; these payments vary according to patient age, and range from 10 % to 30 % of the costs: 20 % for those under 6 years old, 30 % for 6 to 69 year olds, 20 % for 70 to 74 year olds, and 10 % for those over 75 years old.

Patient household income and age regulate the self-payment upper limit, which varies from 77 US dollars (USD) to 1,442 USD per month at the timing of writing, based on the average exchange rate in 2008: 1 USD = 104 Yen (Table 1) [5]. If patients exceed this limit, any overpayments are later reimbursed [5]. The final co-payment is calculated as the total payment minus the amount of the refund. If the patient receives welfare because of having a low income or as a survivor of the atomic bombings of Hiroshima and Nagasaki, his or her medical expenses are fully covered by the government and no out-of-pocket charges are required.

**Table 1:** Reimbursement system for medical expenditures

Age	Income	Self-payment upper limit per month
Under 70 years old	High (monthly income over 530,000 Yen; 5,096 USD)	150,000 Yen (1,442 USD) + [medical expenses – 500,000 Yen (4,808 USD)] × 1 %
	Middle	80,100 Yen (770 USD) + [medical expenses – 267,000 Yen (2,567 USD)] × 1 %
	Low (people who are excluded from inhabitant tax)	35,400 Yen (340 USD)
Over 70 years old	High (monthly income over 280,000 Yen; 2,692 USD)	80,100 Yen (770 USD) + [medical expenses – 267,000 Yen (2,567 USD)] × 1 %, if the patient is hospitalized
		44,400 Yen (427 USD) for outpatient clinic only
	Middle	44,400 Yen (427 USD) , if the patient is hospitalized
		12,000 Yen (115 USD) for outpatient clinic only
	Lower (people who are excluded from inhabitant tax)	24,600 Yen (237 USD) , if the patient is hospitalized 8,000 Yen (77 USD) for outpatient clinic only
	Low (no gross income)	15,000 Yen (144 USD) , if the patient is hospitalized 8,000 Yen (77 USD) for outpatient clinic only

The currency was calculated based on the average exchange rate in 2008: 1 USD = 104 Yen.

Cancer treatment has changed with the development of molecular targeting agents. Specifically, imatinib has dramatically altered the treatment of chronic myelogenous leukaemia (CML) [6,7]. Long-term use of imatinib enables CML patients to live longer in remission [8]. Compared with conventional treatment with interferon-α, imatinib has resulted in fewer side effects and a higher quality of life for CML patients [9,10]. In recent years, it has been become the standard drug treatment for CML, although its cost remains a major problem; 400 mg of imatinib, the standard daily dose, costs 146.1 USD [11].

The costs of cancer treatment have been determined in many studies [12]. However, information on the relationship between patient incomes and the financial burdens of and reactions to expensive therapies is limited. We hypothesised that patients’ income and the financial burden of their treatment could influence their willingness to undergo such medical interventions. In this study, we focused on CML patients who have been prescribed imatinib.

## **Methods**

### **Patients and ethics**

This study was approved by the ethical review board of the Institute of Medical Science of The University of Tokyo (Approval number: 20-70-210417). The survey was conducted during May to August 2009 and included any patients with CML who were prescribed imatinib. In Japan, imatinib mesylate (Glivec®) was approved for coverage by public medical insurance and registered in the National Health Insurance Reimbursement List for the treatment of CML in December 2001.

We asked 345 hospitals across Japan with haematology departments to participate in our survey. Of them, 144 (41.7 %) agreed to participate, and questionnaires were distributed to 1200 CML patients. The patients completed the survey during their regular clinic visits. Nine associations for patients with blood diseases were also asked to take part in our survey. One major CML patient association participated in our survey, while the other eight associations did not. The questionnaires were posted to the patients who belonged to this association and agreed to participate. The questionnaires were completely anonymous and returned by mail. They were sent out on May 1, 2009, and the deadline for response was August 10, 2009. A total of 577 CML patients completed the questionnaire (response rate: 48.1 %). Two patients were excluded from the study because they had never been prescribed imatinib. In total, 575 responses were analysed.

### **Questionnaire and assessment variables**

We prepared a questionnaire with 18 items assessing income, medical costs, and financial burden after a series of discussions among medical staff and economists (Additional file 1: Tables S1 and TableS2). As mentioned above, the questionnaires were distributed to CML patients through their doctors or a patient association. We asked the patients to refer to their previous tax returns when they responded to the questions on income or medical expenses to obtain accurate data.

To assess the financial burden of imatinib prescription, we surveyed the patients’ annual pre-tax household incomes from all sources in 2000, 2005, and 2008, as well as their out-of-pocket medical costs and final co-payments. Household incomes and medical expenses were expressed in Japanese Yen in the questionnaire, while they are in USD in this article. We converted the incomes using the average currency exchange rate of 2008, which was 104 Japanese Yen per USD. Financial burden was based on the subjective views of the patients. The impact of CML and imatinib side effects on patient occupations was also studied. Side effects were based on patient reports.

The patients who were treated with imatinib in 2000 either received the medicine from a clinical trial or they imported it under their physician’s advice. The patients in the clinical trial did not need to pay for the imatinib, but they did pay for other medical costs and received reimbursements for these if they exceeded the ceiling of medical expenditure. The patients who imported imatinib paid for the drug as well for the transaction, and they could not apply to the reimbursement program.

### **Statistical analysis**

Data were analysed using JMP ver. 8.0 (SAS Institute, Inc., Cary, NC) and PASW Statistics 18 (SPSS, Inc., Chicago, IL). All incomes and costs are expressed as annual amounts, which are summarised for each year (2000, 2005 and 2008). To compare the ratio of annual out-of-pocket expenses to household income for these three years, we performed repeated analyses of variance (ANOVA).

A multiple logistic regression analysis was used to determine the variables that independently affected patient consideration to discontinue imatinib treatment, correcting for interactions between the variables. Before the multivariate analysis, univariate analyses were performed with the following variables: age, sex, imatinib dose, the presence of side effects, academic background, occupation, residence of patients with families, annual household income in 2008, out-of-pocket costs, final co-payment, and patient knowledge of medical subsidy programs. The multiple logistic regression analysis was performed after the determination of collinearity between variables that were *p* ≪ 0.25 in the univariate analyses, using the Pearson's correlation coefficient and a generalized linear model. The variables were evaluated with conditional odds ratios (OR) and 95 % confidence intervals (CI). Confidence intervals above 1.0 suggested that the variable was associated with an increased likelihood of considering the discontinuation of imatinib treatment, while those below 1.0 suggested that the variable was associated with a decreased likelihood of considering the discontinuation of imatinib treatment. Confidence intervals that included an OR of 1.0 were not considered statistically significant. Statistical significance was set at *p* ≪ 0.05 for all statistics.

## **Results**

### **Patient backgrounds**

The demographic details of the CML patients are shown in Table 2. The median age was 61 years (range, 15–94), and 428 patients (74.4 %) were ≤ 70 years old. Five patients had no household income and did not receive pensions or other social support; they spent their savings on daily living expenses. A total of 112 CML patients (19.5 %) answered that they have stopped working or been unemployed because of the onset of CML and 34 (5.9 %) have stopped working or been unemployed because of imatinib side effects.

**Table 2:** Demographic and socioeconomic characteristics of the patients

Variables			No. of valid responses (*n*)
Age	Median (range)	61 (15–94)	573
Gender: Men, Women	*n* (%)	369 (64.2), 206 (35.8)	575
Socioeconomic Status			
Household Income (2008) (USD)	Median (range)	39,728 (0–832,000)	433
Occupation			534
Working full-time	*n* (%)	229 (42.9)	
Working part-time	*n* (%)	42 (7.9)	
Homemaker	*n* (%)	2 (0.4)	
Other	*n* (%)	35 (6.6)	
Retired or currently not working	*n* (%)	226 (42.3)	
Education			508
University graduate or higher	*n* (%)	151 (29.7)	
Other	*n* (%)	357 (70.2)	
Living with family: Yes, No	*n* (%)	439 (80.3), 108 (19.7)	547

The currency was calculated based on the average exchange rate in 2008: 1 USD = 104 Yen.

A summary of the patients’ CML treatment and side effects is shown in Table 3. A total of 452 patients (78.6 %) were diagnosed with CML after imatinib sales started in Japan on December 7, 2001. The median period of imatinib administration was 46.5 months (range, 1–115). A total of 525 patients (91.3 %) experienced side effects.

**Table 3:** **Overview of imatinib treatment as of August, 2009 (*n*** **= 575)**

Variables	*n* (%)
Current imatinib dosage	
<400 mg/day	215 (37.4)
400 mg/day	335 (58.3)
>500 mg/day	22 (3.8)
No response	3 (0.5)
Year of CML diagnosis	
Before 1990	4 (0.7)
1991 to 1995	15 (2.6)
1996 to 2000	68 (11.8)
2001 to 2005	242 (42.1)
After 2006	236 (41.0)
No response	10 (1.7)
Number of patients who were treated with imatinib	
2000	68 (11.8)
2005	284 (49.4)
2008	528 (91.8)
Frequency of Visits as an Out-patient	
<1 month	65 (11.3)
More than 1 month, but less than 2 months	320 (55.7)
More than 2 month, but less than 3 months	132 (23.0)
More than 3 month, but less than 4 months	52 (9.0)
No response	6 (1.0)
Side effects*	
Facial oedema	370 (64.3)
Muscle cramps	248 (43.1)
Rash	235 (40.9)
Nausea	214 (37.2)
Fatigue	201 (35.0)
Diarrhoea	180 (31.3)
Muscular pain	141 (24.5)
Generalised oedema	136 (23.7)
Joint pain	80 (13.9)
Taste disorder	67 (11.7)
Stomach pain	49 (8.5)
Other	137 (23.8)

* The question on side effects allowed multiple answers.

### **Patient perspectives on the financial burden of imatinib treatment and discontinuation of the prescription**

In 2000, 28 patients out of the 68 who were given an imatinib prescription (41.2 %) felt that their medical expenses constituted a heavy financial burden, although not all medical expenses reflected the cost of imatinib because imatinib was not approved in Japan in 2000. This number and ratio have increased more recently, rising to 201 out of 284 (70.8 %) in 2005, and 400 out of 528 (75.8 %) in 2008.

**Figure 1:**
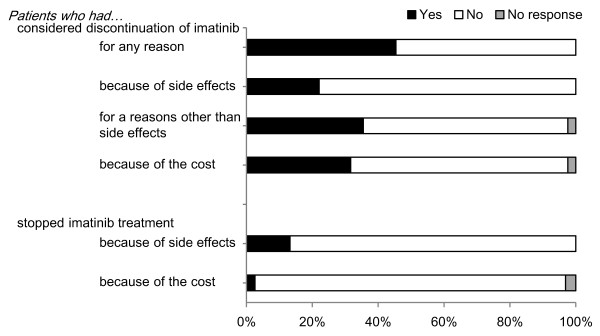
**Patient perspectives on the discontinuation of imatinib treatment.** A bar chart of patient perspectives on the discontinuation of imatinib treatment is shown. The proportion of patients indicating each reason is shown on the X axis.

A total of 261 patients (45.4 %) had considered discontinuing their imatinib prescription; 127 (22.1 %) because of its side effects and 204 (35.5 %) because of other reason(s) (Figure 1). A total of 182 patients (31.7 %) considered its discontinuation because of the financial burden that its use created. Seventy-six patients (13.2 %) actually stopped their imatinib prescription. Of them, 40 (7.0 %) stopped based on their physicians’ advice because of the side effects, 18 (3.1 %) stopped of their own accord because of the side effects, and 18 (3.1 %) stopped for other reasons, e.g., to prepare for surgery, or because there was no treatment response. Imatinib treatment was temporarily suspended by 15 (2.6 %) patients because of financial reasons.

### **Financial situation and medical expenses in 2000, 2005 and 2008**

**Figure 2:**
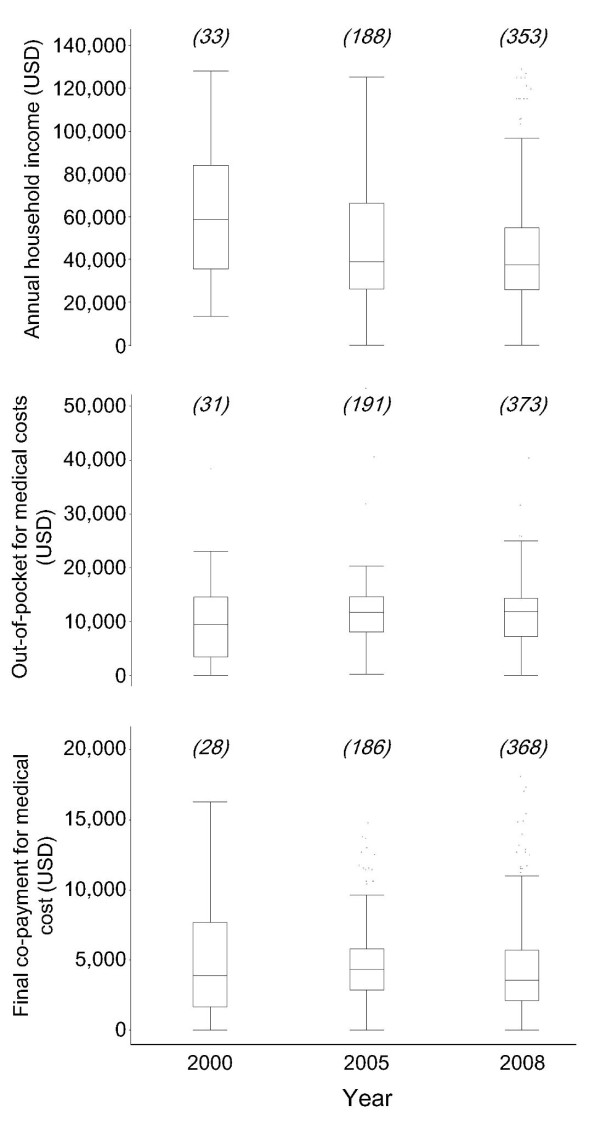
**Annual incomes and medical costs of CML patients.** Box plots of medical expenses and incomes by study year are shown. The number of valid responses for each year is indicated on the upper row of each box. The box plots represent the median, 25th and 75th percentiles (the box) as well as the range without outliers (the whiskers). The outliers were defined as 1st or 3 rd quartile ± 1.5 × interquartile range. The currency was calculated based on the average exchange rate in 2008:1 USD = 104 Yen.

Patients’ annual household incomes have dropped since 2000; however, their out-of-pocket expenses and final co-payments have remained unchanged (Figure 2). The changes in median household income, out-of-pocket expenses and final co-payments between 2000 and 2008 were −21,058, +2,212 and −337 USD, respectively. Five patients who had no household income spent their savings on medical expenses.

**Figure 3:**
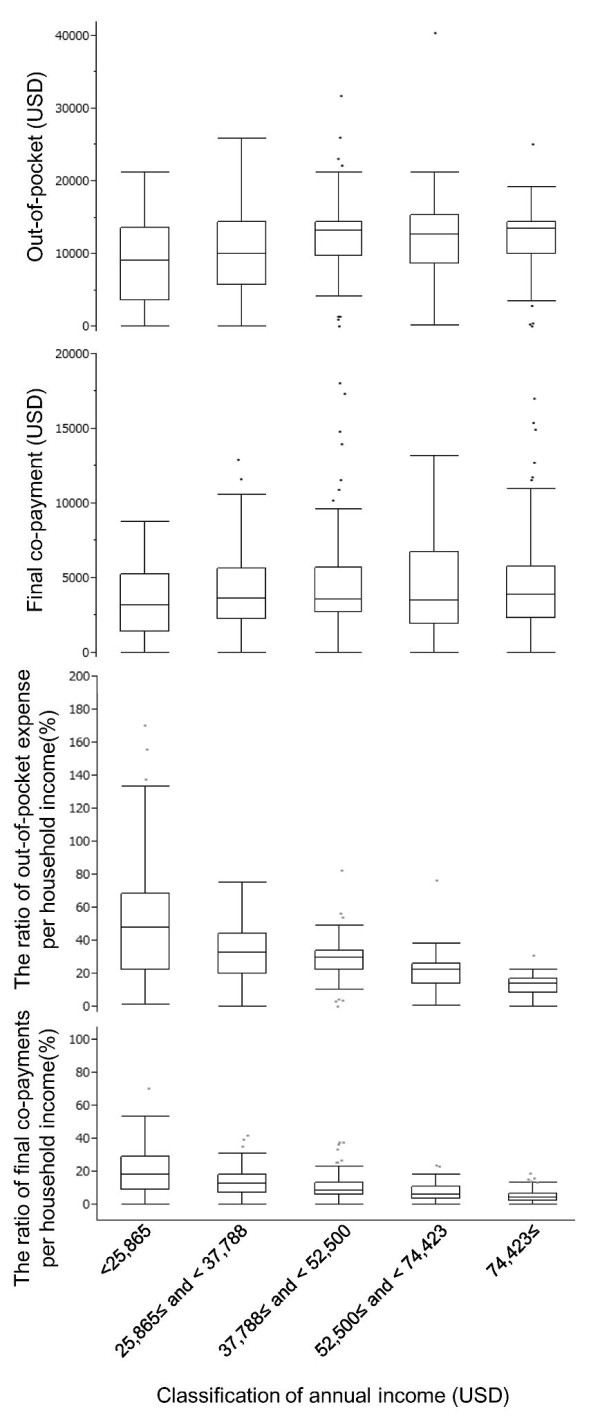
**Medical expenses in 2008 by quintile groups of annual income.** The classification of annual income on the X axis is based on the 2008 annual report of family income and expenditure in Japan. The currency was calculated based on the average exchange rate in 2008:1 USD = 104 Yen. The box plots represent the median, 25th and 75th percentiles (the box) as well as the range without outliers (the whiskers). The number of valid answers for each income group was 93, 96, 79, 41 and 59 across smaller to larger X axis categories.

The relationships between 2008 household incomes and out-of-pocket expenses and final co-payments are shown in Figure 3. There was no clear correlation between final co-payments and income (*p* = 0.40). Higher ratios of out-of-pocket expenses and final co-payments were observed in patients with lower household incomes.

### **Financial situations of patients who were treated with imatinib throughout 2000 to 2008**

In all, 33 patients answered that they were treated with imatinib throughout the study period; that is, from 2000 to 2008. Among them, 22 were analysed after excluding 11 patients whose medical insurance had changed during the study period. The ratios of annual out-of-pocket expenses for medical costs to annual household income [median, (range)] were 16.8 % (range, 0.0–85.9), 18.4 % (range, 0.0–72.0), and 22.0 % (range, 0.0–120.0) in 2000, 2005 and 2008, respectively. No significant differences were found between the three years in a repeated ANOVA (*p* = 0.23). The ratio of out-of-pocket expenses to household income in 2005 increased for 11 patients and decreased for another 11 compared with the ratio in 2000. When comparing the same ratio for 2005 and 2008, an increase for 13 patients and a decrease for seven patients occurred. The ratio of out-of-pocket expenses did not change for two patients.

### **Factors considered in the discontinuation of imatinib**

The results of the univariate analyses performed to identify the factors associated with patients’ having considered discontinuing their imatinib prescription are shown in Additional file 1: Table S3. Variables with a *p* value ≪ 0.25 in the univariate models were selected as candidates for the multivariate regression analyses. Final co-payments for medical expenses in 2008 and out-of-pocket medical expenses in 2008 were significantly correlated (Pearson’s *r* = 0.810 and *p* ≪ 0.001). Therefore, to avoid collinearity, only final co-payment was selected for further analysis. Both education and occupation showed significant impacts on household income in 2008 (both *p* values ≪ 0.001 in a generalized linear model), and household income was selected for the multivariate model even though its univariate *p* value was ≫ 0.25. Consequently, five factors were considered in the multivariate regression model. Among them, the following three factors were statistically significant predictors of having considered discontinuing imatinib treatment (Table 4): age (OR = 0.96, 95 % CI: 0.95–0.98, *p* ≪ 0.0001), household income (per 10,000 USD) (OR = 0.92, 95 % CI: 0.85–0.99, *p* = 0.03), and final co-payments for medical expenses (per 10,000 USD) (OR = 2.21, 95 % CI: 1.28–4.28, *p* = 0.004). The 95 % CIs for these three variables do not include an OR of 1, thus we conclude that the likelihood of having considered discontinuing imatinib treatment decreased with increasing patient age and income, and increased with final co-payments for medical expenses.

**Table 4:** Factors associated with patient consideration to discontinue imatinib treatment for a reason other than side effects

Variables	Tested value	Odds ratio (95 % CI)	*p* value
Age	One year of increased age	0.96 (0.95–0.98)	≪0.0001
Daily imatinib dose	100 mg of increased dose	0.97 (0.73–1.29)	0.83
Residence with family	-	1.02 (0.81–1.28)	0.89
Household income in 2008	per 10,000 USD increase	0.92 (0.85–0.99)	0.03
Final co-payments for medical expenses in 2008	per 10,000 USD increase	2.21 (1.28–4.28)	0.004

Variables that were included in the multiple regression model identifying factors associated with patient consideration to discontinue imatinib for a reason other than side effects are shown.

## **Discussion**

This is the first study to demonstrate that the use of imatinib places a large financial burden on Japanese CML patients, who live in a nation with universal health insurance coverage. In countries where patients must pay a part of their medical expenses, such as Japan, those who require expensive medications incur an enormous financial burden. For example, out-of-pocket expenses and final co-payments among patients receiving imatinib in 2008 were approximately 12,000 and 3,600 USD, respectively. Their annual out-of-pocket expenses were about 10 times those of an average healthy Japanese person [13]. These medical expenses continue for as long as the patients are prescribed imatinib, creating an economic problem that is also observed in many other developed countries [14].

Of the 204 patients who had considered discontinuing their imatinib treatment for reasons other than side effects, 182 (90 %) had considered it for financial reasons. The proportion of patients who felt that the financial burden was getting higher every year was approximately 40 % in 2000 and 76 % in 2008. The proportion of patients who actually stopped their imatinib prescription for financial reasons was 2.6 %. In a US study, 31 % of CML patients stopped their imatinib treatment [15]. It should be noted that Japan has a universal health insurance system that guarantees all Japanese citizens access to any hospital and standard therapies that are regulated by the national government, regardless of their income or type of private medical insurance. These findings suggest that having a sense of financial burden might affect patients’ compliance with continuing to take the high-cost medication, even though they can change their hospitals easily to keep up the medication in cases of conflict between patients and hospital staff. Therefore, the provision of medical insurance programs specifically for patients with severe economic hardship who require costly medications should be considered.

This study showed that many CML patients had insufficient financial resources to pay for their imatinib treatments. The average annual household income in Japan in 2008 was 55,000 USD [13], which vastly exceeds that of this study’s CML patients (37,500 USD) (Figure 2). In all, 13 % of patients were in the low-income group with a household income of 22,000 USD or less [16]. Furthermore, patients’ financial situations are worsening; household income in Japan dropped by an average of 6,673 USD between 2000 and 2008 [17] while the median income of the CML patients fell by approximately 13,000 USD in the same period (Figure 2). It will become increasingly difficult for the patients to continue paying for their medical expenses. Because imatinib was not approved for sale in Japan until 2001, the data on medical expenditures in 2000 should be interpreted with caution. Unknown bias may be present because of to the small population of patients who were treated with imatinib in 2000, as well as the unequal co-payment/reimbursement conditions in 2000 versus 2005 and 2008. The data on medical costs in 2000 may be affected by these differences in co-payment/reimbursement conditions.

There are several possible reasons why the financial situations of the CML patients have deteriorated. One explanation may be that their household income has decreased because of the onset of unemployment after their diagnosis of CML. In our study, 112 CML patients, who accounted for 20 % of the total valid responses, had quit their jobs because of the onset of CML, although the unemployment rate in this study is lower than that reported among patients with other malignant diseases [18]. Even if they could obtain new jobs to adjust their working conditions to their treatment, some of them may be re-employed at a lower wage. Another reason may be that the on-going recession in Japan caused the elevated unemployment rate, because the poor economy makes it difficult for cancer patients to find new jobs or to return to their former ones. One must also mention the side effects of imatinib as a third factor. Most physicians believe that imatinib has minimal side effects and that the patients can live normal lives during treatment [7]. The symptomatic side effects reported in this study are similar to those indicated in the national registry of the Pharmaceutical Medical Devices Agency of Japan, although we could not collect confirmatory laboratory data [19]. However, the actual lifestyles of the patients were quite different from physicians’ expectations; no fewer than 6.0 % of the patients had to quit their jobs because of imatinib side effects, with the majority of them unable to return to their workplaces, suggesting that side effects have a greater impact on patients’ careers than is evident in the medical literature. Another possible reason is retirement during the study period, because patients aged between 56 and 73 years old in 2009 may have retired during the period of interest.

The multivariate analysis showed that household income, total medical expenses, and age affected the consideration to discontinue imatinib treatment. Younger patients and patients with lower incomes were more likely to have considered discontinuing their imatinib treatment, whereas patients with higher incomes were less likely to have considered discontinuing their treatment (Table 4). In addition, 25.3 % of our cohort had incomes of 25,000 USD or less in 2008, and the median ratio of their out-of-pocket expenses to their incomes was approximately 50 % (Figure 3). Thus, low-income patients face great difficulty in pursuing normal lives in the long-term.

Supplemental support for low-income patients who are not covered by social security and are being treated with expensive drugs for a long term should be considered, because the current refund system was designed for high medical expenses with short-term duration, such as surgical operations, not for new high-cost drugs for the treatment of cancer or rheumatoid arthritis. Although the development of a new support system for low-income patients would bring about additional cost to the government, we believe the system is still feasible, because it was reported that non-adherent CML patients prescribed imatinib might be more costly to the health care system than adherent patients [15].

The CML patient association has proposed a method to reduce medical expenses among its members. Since the summer of 2007, the association has informed members of the benefits of a three-month imatinib prescription. The three-month prescription is beneficial because refunds are calculated on a monthly basis and the maximum limit of the refunds is not affected by medical costs, meaning that prescribing imatinib in a particular month as much as possible can bring higher patient refunds. The refund system can be applied when patients pay medical expenses over the maximum limit four times or more per year; thus, a three-month prescription is the most reasonable way to reduce annual final co-payments. This information was shared with CML patients and might be related to the reduction of final co-payments in 2008 by 800 USD compared with 2005 (Figure 2). Proposing imatinib subscriptions every three months increases the payment of each clinic visit by about 1,923 USD; however, the annual final co-payment is reduced by approximately 3,846 USD because the patients can obtain larger annual refunds compared with those following monthly prescriptions. A similar movement by patient associations to decrease the financial burden of imatinib exists in Korea [20]. Unfortunately, not all patients have been able to reduce their annual final co-payment using three-month imatinib prescriptions. In fact, 49.6 % of patients continue to visit hospitals every month, even though their CML symptoms show no signs of worsening. It is possible that these patients regularly visit hospitals to reduce their medical expenses for each visit, even though a three-month interval between visits is sufficient from a medical standpoint [21].

From the perspective of patient accessibility, the Japanese universal health insurance system is beneficial because it guarantees that patients can access any hospital/clinic. However, financial difficulties among low-income patients who are treated with expensive medicines might affect the accessibility of long-term advanced medication, suggesting a limitation of the current universal insurance system in Japan. Health care reform to support low-income patients is required.

Dasatinib and nilotinib, which are newly developed tyrosine kinase inhibitors for CML, were approved for use in Japan in March 2009 and March 2011, respectively [22,23]. The prices of these medicines regulated by the national institute for approved dosage per day are as follows: dasatinib (100 mg) 18,448 Yen and nilotinib (800 mg) 18,428 Yen [24]. For reference, that of imatinib (400 mg) is 10,996 Yen. The patients treated with dasatinib or nilotinib can also receive reimbursement if their medical expenses exceed the upper limit of co-payment.

There are several limitations to this study. To begin with, it is a small-cohort, retrospective survey that relies on a questionnaire. Income and medical expenses were simply based on patient responses, although we did ask the patients to refer to their previous tax-return documents to obtain accurate data. The other limitation is that this study included only Japanese patients, and the social system, culture, and patient backgrounds of Japan are likely to have affected our findings. For example, 37.4 % of the patients in our survey were prescribed an imatinib dose of 300 mg/day or less. This dose level, as well as the age and gender composition of our participants, is similar to previous reports from Japan [9,25], but the dose is less than that reported in similar studies in the United States and Europe [8]. Importantly, the health insurance system in Japan is different from other countries, meaning that the flow of medical expenditures and refunds in the household budget is different. Thus, careful consideration is needed when comparing the financial burdens of imatinib internationally.

## **Conclusions**

In this report, we showed that the proportion of CML patients who sensed a financial burden increased between 2000 and 2008, meanwhile their annual incomes fell by 13,000 USD even their medical expenses did not change. The findings presented here suggests that financial support for those patients being treated with expensive drugs still remains a major problem in Japan. This study is a small-cohort, retrospective survey, however, issues such as aging populations, excessive budget deficits, the high prices of newly developed medications, and elevated out-of-pocket expenses are common problems in developed countries. These issues must be overcome; otherwise, newly developed, revolutionary drugs will not be able to bring benefits to large numbers of patients. Detailed discussions of public financial support are needed to reduce the financial burden on patients.

## **Competing interests**

The authors declare no conflicts of interest in this study.

## **Authors’ contributions**

YK, RM designed the research, wrote the paper, and analysed and managed the data. TM, YK, NM, YT, and NH carried out data collection and reviewed the paper. AM and MK reviewed the paper and gave final approval. MT contributed to revision of the paper. All authors approved the final version of the paper for submission.

## Pre-publication history

The pre-publication history for this paper can be accessed here:

http://www.biomedcentral.com/1471-2407/12/152/prepub

## Supplementary Material

Additional file 1 **Table S1. **Table S1. Questionnaire Form 1, **Table S2.** The questionnaire form 2. Patients filled in the following table on their incomes and medical costs in units of 10,000 yen. Final co-payments were calculated as medical expenses minus the refunds, **Table S3.** The univariate regression analysis to determine variables associated with patient consideration of discontinuing imatinib treatment for a reason other than side effects. Click here for file
