# Supplementary material for: Increased financial burden among patients with chronic myelogenous leukaemia receiving imatinib in Japan: a retrospective survey
Source: BMC Cancer. 2012 Apr 24;12:152. doi: 10.1186/1471-2407-12-152 (PMC3502305; doi:10.1186/1471-2407-12-152)
Supplement: Additional file 1 — Table S1. Table S1. Questionnaire Form 1, Table S2. The questionnaire form 2. Patients filled in the following table on their incomes and medical costs in units of 10,000 yen. Final co-payments were calculated as medical expenses minus the refunds, Table S3. The univariate regression analysis to determine variables associated with patient consideration of discontinuing imatinib treatment for a reason other than side effects. [file 1471-2407-12-152-S1.doc]

Supplemental Table 1. Questionnaire Form 1

| Question number | Question | Your Answer |
| --- | --- | --- |
| 1 | Please fill in your birth year. | _ (Year) |
| 2 | When were you diagnosed with chronic myelogenous leukaemia? | _/_ (Year/Month) |
| 3 | What is your gender? | □Man, □Woman |
| 4 | We would like to ask you to fill in your educational background. Please check the most appropriate item. | □Junior high school,  □High school,  □College or university  □Graduate school or higher level of school |
| 4 | Do you know the subsidy program for expensive medical expenses? | □Yes, □No |
| 5 | Please fill in the following table on your income and medical expense. | (See supplemental table 2) |
| 6 | How often do you visit your doctor? | □Every week,  □Every two weeks,  □Every three weeks,  □Every month,  □Every two months  □Every three months  □Other (Please specify the frequency of your clinic visit:_) |
| 7 | Have you ever had imatinib treatment? | □Yes, □No |
| 8 | When did you start your imatinib treatment? | _/_ (Year/Month), or □N/A |
| 9 | How many imatinib tablets do you currently take? | _ tablets per day |
| 10 | Have you ever had the side effects of imatinib? | □No,  □Rash,  □Nausea,  □Muscle cramps,  □Generalised oedema,  □Facial oedema,  □Fatigue,  □Diarrhoea,  □Joint pain,  □Muscular pain,  □Taste disorder,  □Stomach-ache  □Other (Please specify:_) |
| 11 | Have you ever stopped imatinib treatment because of side effects? | □Yes, □No |
| 12 | Have you ever stopped imatinib treatment for a reason other than the side effects? | □Yes (Please go to question #13),  □No (Please go to question #14) |
| 13 | Why did you stop imatinib? | □Doctor’s advice,  □Self-judgment  □High medical expense  □I did not want to rely on imatinib  □I just did not want to have imatinib  □Other (Please specify:_) |
| 14 | Have you ever considered discontinuing imatinib treatment? | □Yes (Please go to question #15),  □No (Please go to question #16) |
| 15 | Why did you considered discontinuing imatinib treatment? | □High medical expense  □I did not want to rely on imatinib  □I just did not want to have imatinib  □Other (Please specify:_) |
| 16 | We would like to know your working condition. Please check the most appropriate items. | □Working full time  □Working part time  □Homemaker  □Other  □Retired or currently not working (Please go to question #17 and #18) |
| 17 | Have you ever stop working or been unemployed because of the onset of chronic myelogenous leukaemia? | □Yes, □No |
| 18 | Have you ever stop working or been unemployed because of the side effects of imatinib? | □Yes, □No |

Supplemental Table 2. The questionnaire form 2

Patients filled in the following table on their incomes and medical costs in units of 10,000 yen. Final co-payments were calculated as medical expenses minus the refunds.

| Year | 2008 | 2005 | 2000 |
| --- | --- | --- | --- |
| Annual household income (units of 10,000 yen) |  |  |  |
| Annual medical expenses that you paid to hospitals, clinics or pharmacies (units of 10,000 yen) |  |  |  |
| Annual refunds for medical expenses (units of 10,000 yen) |  |  |  |
| Do you feel the financial burden of your medical expenses? | □Yes, □No | □Yes, □No | □Yes, □No |

Supplemental Table 3. The univariate regression analysis to determine variables associated with patient consideration of discontinuing imatinib treatment for a reason other than side effects

| Variables | Tested value | Odds ratio | 95% CI | *p* value |
| --- | --- | --- | --- | --- |
| Age | One year of increased age | 0.97 | 0.96-0.98 | < 0.0001 |
| Gender | Men | 0.89 | 0-61-1.29 | 0.54 |
| Daily imatinib dose | 100 mg of increased dose | 1.15 | 0.95-1.41 | 0.15 |
| Side effects | No presence of side effects | 0.74 | 0.37-1.49 | 0.40 |
| Education | University graduate or higher | 0.73 | 0.48-1.11 | 0.14 |
| Occupation | Retired or currently not working | 1.36 | 0.93-2.00 | 0.11 |
| Residence with family | - | 0.71 | 0.47-1.07 | 0.10 |
| Household income in 2008 | per 10,000 USD increased | 0.97 | 0.92-1.02 | 0.23 |
| Final co-payments for medical expenses in 2008 | per 10,000 USD increased | 1.00 | 1.00-1.00 | 0.03 |
| Out-of-pocket medical expenses in 2008 | per 10,000 USD increased | 1.18 | 0.91-1.54 | 0.21 |
| Knowledge of medical subsidy program | - | 1.13 | 0.68-1.89 | 0.64 |
